# Significance of Normal Lung Volume on Quantitative CT Imaging Analysis in Group 1 and Group 3 Pulmonary Hypertension

**DOI:** 10.1016/j.chpulm.2024.100062

**Published:** 2024-05-11

**Authors:** Tadasu Okaya, Ayako Shigeta, Nobuhiro Tanabe, Koichiro Tatsumi, Hajime Yokota, Akira Nishiyama, Akira Naito, Ayumi Sekine, Toshihiko Sugiura, Seiichiro Sakao, Takuji Suzuki

**Affiliations:** a Department of Respirology, Graduate School of Medicine, Chiba University, Chiba, Japan; b Department of Radiology, Graduate School of Medicine, Chiba University, Chiba, Japan; c Department of Respiratory Medicine, Chibaken Saiseikai Narashino Hospital, Chiba, Japan; d Department of Radiology, Tsudanuma Central General Hospital, Chiba, Japan; e Department of Respiratory Medicine, Graduate School of Medicine, International University of Health and Welfare, Chiba, Japan

**Keywords:** CT imaging, parenchymal lung diseases, pulmonary hypertension, quantitative CT

## Abstract

**Background:**

Patients with concurrent pulmonary hypertension (PH) and parenchymal lung diseases have a high risk of mortality. However, whether their outcomes are related to low normal lung volume (NLV) resulting from parenchymal lung diseases on chest high-resolution CT (HRCT) imaging remains unknown.

**Research Question:**

Would NLV on quantitative HRCT imaging affect disease behavior in patients with PH?

**Study Design and Methods:**

This retrospective observational study evaluated patients with physician-diagnosed group 1 and group 3 PH among 1,471 patients who underwent right heart catheterization. Using a 3-dimensional image analysis system for HRCT imaging, the percentage of NLV (–950 to –600 Hounsfield units) to the whole lungs (%NLV) was calculated. The optimal cutoff point of %NLV for predicting survival was examined using the receiver operating characteristic (ROC) curve. The Kaplan-Meier method and Cox proportional hazards regression were used to detect the association between %NLV and prognosis. Multivariable logistic regression was performed to examine the association of %NLV with response to pulmonary vasodilators.

**Results:**

Overall, 157 patients (mean age, 53.1 ± 17.6 years; sex, n = 111 [70.7%] female patients) were included. ROC curve analysis showed that the optimal cutoff of %NLV for predicting survival was 83.2%. The patients with %NLV of ≥ 83.2% showed significantly higher 5-year survival than that of those with %NLV of < 83.2% (81.7% vs 36.6%; *P < .*0001). Multivariable logistic regression analysis revealed %NLV of < 83.2% as an independent prognostic factor (hazard ratio, 2.49 [95% CI, 1.14–5.44]; *P = .*022). Responders showed significantly higher %NLV than nonresponders (90.0 ± 5.1% vs 84.7 ± 9.2%; *P = .*0002). Multivariable regression analysis showed that only high %NLV predicted response (OR, 1.12 [95% CI, 1.01–1.23]; *P = .*016).

**Interpretation:**

Quantitative CT imaging analysis might allow numerical quantification of the lung condition and vasodilator-treatable area beyond subjective visual assessment in patients with PH. The %NLV could be a novel predictor of prognosis and treatment response in these patients.

**Take-home Points**

**Study Question:** Would normal lung volume on quantitative CT imaging affect the outcomes of patients with pulmonary hypertension (PH)?

**Results:** Multivariable regression analysis revealed the percentage of normal lung volume of < 83.2% as an independent prognostic factor (hazard ratio, 2.49 [95% CI, 1.14-5.44]; *P = .*022), and a high percentage of normal lung volume was the only predictor of response to pulmonary vasodilators (OR, 1.12 [95% CI, 1.01-1.23]; *P = .*016).

**Interpretation:** The percentage of normal lung volume could be a novel predictor of prognosis and treatment response to pulmonary vasodilators in patients with PH.

Pulmonary hypertension (PH) is an intractable disease characterized by elevated pulmonary arterial pressures. PH is classified into five clinical subgroups based on cause and pathophysiologic characteristics.^1^ The treatment response to pulmonary vasodilators and prognosis differ among the five subgroups.^2^ Specifically, the prognosis of patients with group 1 PH, characterized by idiopathic pulmonary arterial hypertension (PAH), has been improved remarkably by continuous IV prostaglandin I2 therapy and upfront combination therapy of pulmonary vasodilators.^3^, ^4^, ^5^ In contrast, patients with group 3 PH resulting from parenchymal lung diseases such as COPD or interstitial pneumonia rarely benefit from pulmonary vasodilators.^6^ Moreover, the prognosis of patients with group 3 PH remains poorer than that of those with group 1 PH.^1^^,^^2^^,^^7^

Parenchymal lung disease could be observed on chest CT imaging in patients with PH. However, the degree of parenchymal lung disease in patients with PH in relationship to prognosis remains objectively undefined. Therefore, definitive diagnosis of either group 1 or group 3 PH is challenging, especially when mild parenchymal lung disease exists. Patients with intermediate features between group 1 and group 3 PH recently were identified. Among patients with group 3 PH, some patients have a cardiac index of < 2.0 L/min/m^2^ or mean pulmonary artery pressure of ≥ 35 mm Hg, referred to as the PAH phenotype. These patients could respond to pulmonary vasodilators.^8^ Moreover, patients with idiopathic PAH with the lung phenotype, that is, disease defined by smoking history and diffusing capacity of the lungs for carbon monoxide (Dlco) of < 45% predicted, were reported to have a worse survival rate than that of patients with classical idiopathic PAH.^9^ Therefore, a useful new indicator of the degree of parenchymal lung disease is required for phenotyping and predicting the treatment response and prognosis in patients with PH.

Chest high-resolution CT (HRCT) imaging can reflect pathologic changes in the lungs and can reveal the morphologic features of parenchymal lung diseases. In patients with group 3 PH, emphysematous and interstitial fibrotic changes are observed on HRCT imaging. In contrast, patients with group 1 PH theoretically show almost normal lung density on HRCT imaging. Based on these findings, when categorizing patients with PH accompanied by parenchymal lung diseases into group 1 or group 3 PH, a visual interpretation of lung parenchymal changes on HRCT imaging commonly is used.^10^ Moreover, in patients with PAH, the presence of lung parenchymal change on HRCT imaging was reported to be an independent poor prognostic factor.^11^^,^^12^ Thus, the evaluation of lung parenchymal changes on HRCT imaging is considered to be important for patients with PH with concurrent parenchymal lung diseases; however, to the best of our knowledge, no study has focused on and investigated the degree of normal structures in the lungs. In addition, the significance of a normal lung volume (NLV) on HRCT imaging and its outcomes in PH remains unknown.

Thus, this study aimed to clarify the association between NLV on quantitative CT imaging and the prognosis of patients with PH and to investigate the relationship between quantitative NLV and the response to pulmonary vasodilators. We focused on NLV in the quantitative CT imaging analysis of patients with PH and hypothesized that patients with higher amounts of NLV would have better outcomes.

## Study Design and Methods

**Figure 1:**
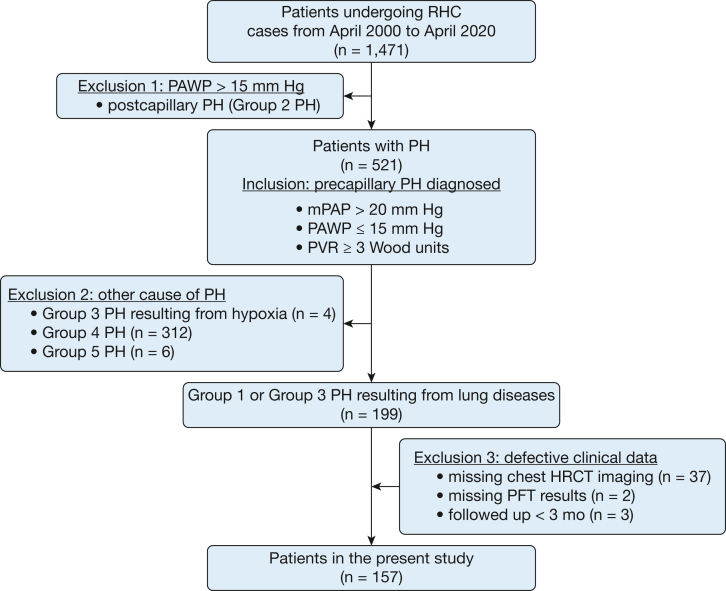
Flow chart showing patient selection. A total of 157 patients were included in the present study based on the inclusion and exclusion criteria. HRCT = high-resolution CT; mPAP = mean pulmonary arterial pressure; PAWP = pulmonary arterial wedge pressure; PFT = pulmonary function testing; PH = pulmonary hypertension; PVR = pulmonary vascular resistance; RHC = right heart catheterization.

This was a retrospective, single-center, observational cohort study of consecutive patients who underwent right heart catheterization (RHC) between April 2000 and April 2020 and were recruited from a registry at Chiba University Hospital, Chiba, Japan. Among the 1,471 patients identified, 521 patients with a diagnosis of precapillary PH (excluding patients with isolated postcapillary PH or combined precapillary and postcapillary PH) according to the Sixth World Symposium on Pulmonary Hypertension were evaluated (Fig 1). Based on the cause of PH, patients with group 3 PH resulting from hypoxia, group 4 PH, or group 5 PH were excluded, and patients with group 1 PH and group 3 PH resulting from lung diseases diagnosed by physicians who specialized in PH were included. In addition, we excluded patients who did not undergo either chest HRCT imaging or pulmonary function testing (PFT) at baseline RHC and those who could not be followed up for > 3 months. This study was approved by the Research Ethics Committee of Chiba University School of Medicine (Identifier: M10403).

### Data Collection

Patient characteristics at the time of baseline RHC were collected from electronic medical records. These included age, sex, BMI, World Health Organization functional classification, Brinkman index score, use of oxygen therapy, classifications of HRCT imaging interpretation, and PH subgroups. Information regarding pulmonary vasodilators also was obtained. The following examinations were performed at baseline RHC: blood test, PFT, 6-min walk test (6MWT), ultrasound cardiography, and RHC.

### Classification of HRCT Imaging Interpretation and PH Subgroups

Regarding interpretation of chest HRCT imaging by two experienced respiratory physicians, the degree of lung parenchymal change was classified visually into the following groups: none to minimal, < 10% lung parenchymal change; intermediate, 10% to 50%; and extensive, > 50%. The patients were divided into the following three PH subgroups based on clinical characteristics: group 1 PH, group 1 plus 3 PH, and group 3 PH. The group 1 plus 3 PH included patients with both PAH features and parenchymal lung diseases.

### Quantification of NLV on Chest CT Imaging

**Figure 2:**
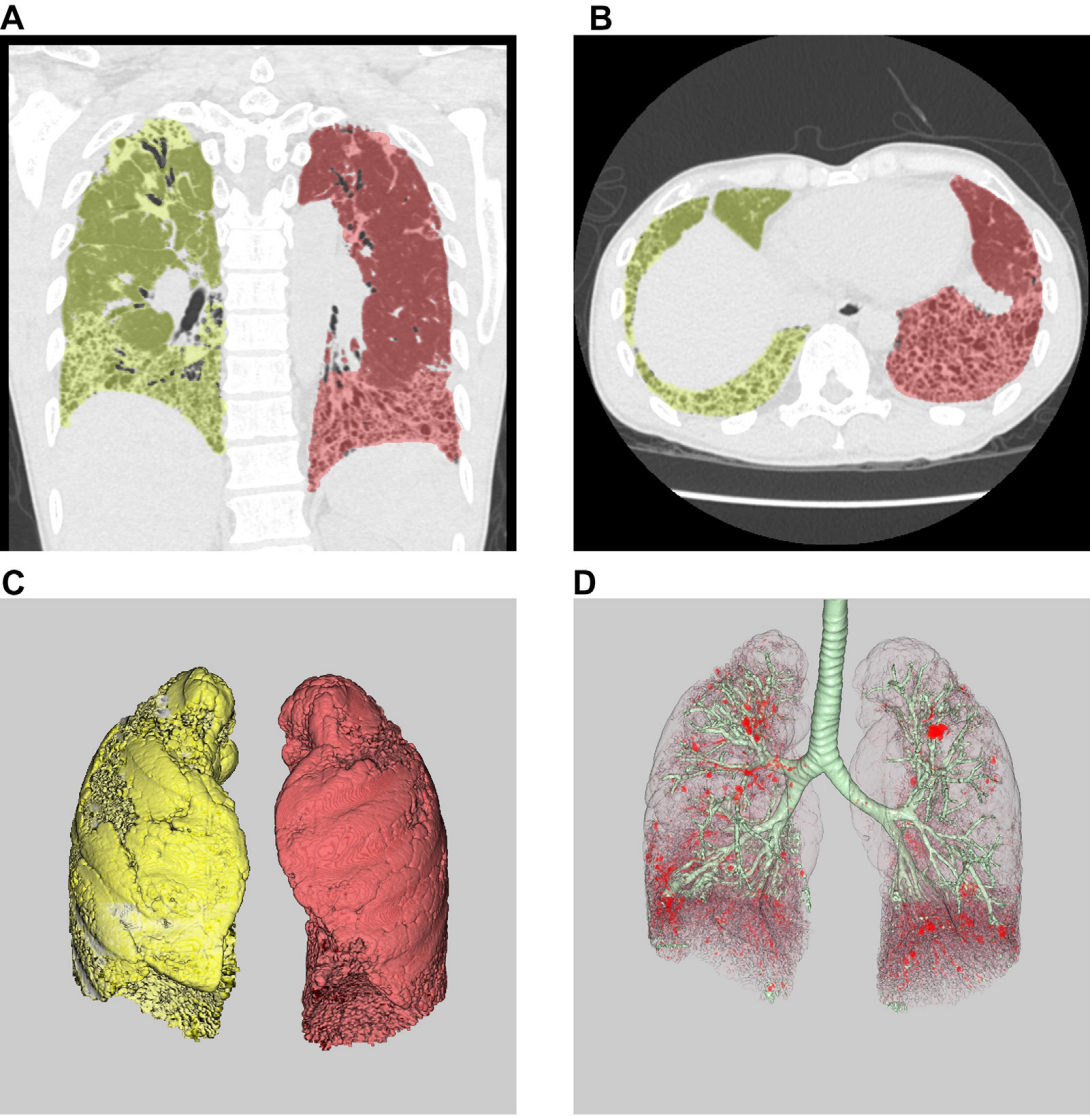
Analysis of quantitative normal lung volume on chest high-resolution CT (HRCT) imaging using a 3-dimensional image analysis system (Synapse Vincent version 6.1; Fujifilm Corporation). A-C, The lung areas on HRCT imaging are identified—coronal section (A) and axial section (B)—and the total lung volume is abstracted and synthesized (C). D, The red spaces in the lungs represent abnormal areas identified by –1024 to –950 Hounsfield units (HU), and the areas of –950 to –600 HU are defined as normal lung volume.

The chest HRCT images of the patients at the time of baseline RHC were analyzed using a 3-dimensional image analysis system (Synapse Vincent version 6.1; Fujifilm Corporation) (Fig 2). Lung fields on HRCT imaging were identified using Hounsfield units (HU) of –1,024 to –200 HU. To distinguish the areas of normal lung parenchyma from those of abnormal lung parenchyma on chest CT scan, areas of –950 to –600 HU were defined as NLV, whereas areas of –1,024 to –950 HU and –600 to –200 HU were regarded as lung parenchymal changes of emphysema and fibrosis, respectively, based on previous studies.^13^^,^^14^ The percentage of NLV to the whole lungs (%NLV) was calculated as the quantitative NLV. With this approach, patient characteristics with sufficient or less %NLV specifically were as follows: those with sufficient %NLV were represented as young female patients with PAH who have no history of smoking or dust exposure, whereas those with less %NLV most often were represented as older adult male patients with PH associated with combined pulmonary fibrosis and emphysema who have a history of heavy smoking.

### Statistical Analysis

Continuous variables are summarized as the mean ± SD or median (interquartile range), whereas categorical variables are presented as absolute numbers and percentages. A histogram was used to express the distribution of %NLV, and stratified histograms with the classifications of HRCT imaging interpretation and PH subgroups are presented. The association between %NLV and PFT parameters is shown in scatterplots, and the correlation coefficients were calculated. The survival rates were examined from the date of baseline RHC until death, loss to follow-up, or April 1, 2021. For survival analysis, data were censored at the point at which the patient died or was no longer reachable because of transfer or self-interruption of hospital visits.

Receiver operating characteristic (ROC) curves were generated to examine the area under the ROC curve of %NLV for 3-year survival and to investigate the optimal cutoff point of %NLV. Five-year survival curves separated by the cutoff point of %NLV and severe ventilatory impairment were generated using the Kaplan-Meier method and were compared between groups using the log-rank test with Bonferroni correction. Severe ventilatory impairment was defined as either FVC of < 70% predicted or FEV_1_ of < 60% predicted.^10^ Cox proportional hazards regression analyses were conducted to detect the hazard ratios (HRs) of %NLV, severe ventilatory impairment, age, sex, and covariates associated with a poor prognosis for PAH and group 3 PH.^15^, ^16^, ^17^ Likelihood ratio tests were performed to examine the goodness of fit.

Data of patients who received pulmonary vasodilators and underwent either follow-up RHC or the 6MWT were extracted, and their responses to pulmonary vasodilators were evaluated. Responders to pulmonary vasodilators were defined as those who showed a decrease in pulmonary vascular resistance of ≥ 15% at follow-up RHC or increase in 6MWT distance of ≥ 15% at follow-up.^18^ The differences between responders and nonresponders were assessed using the Mann-Whitney *U* test or χ^2^ test as appropriate. Logistic regression analysis was performed to examine the OR and 95% CI for responders, and likelihood ratio tests were performed to assess the goodness of fit. All statistical analyses were performed using JMP Pro version 16.1.0 software (SAS Institute, Inc.). Statistical significance was set at a two-sided *P* value of .05.

## Results

### Patient Characteristics

Overall, 157 patients were included (Fig 1). The median follow-up period was 38 months (range, 20.5-89.5 months). The patient characteristics are shown in Table 1. The mean age was 53.1 ± 17.6 years, and 111 patients (70.7%) were female. With respect to the classification of HRCT imaging interpretation of lung parenchymal change, 100 patients (63.7%) showed none to minimal findings, 22 patients (14.0%) showed intermediate findings, and 35 patients (22.3%) showed extensive findings. For PH classification, 89 patients (56.7%) received a diagnosis of group 1 PH, 27 patients (17.2%) received a diagnosis of group 1 plus group 3 PH, and 41 patients (26.1%) received a diagnosis of group 3 PH. A total of 130 patients (82.8%) were treated with pulmonary vasodilators during the follow-up period, and the most common ones used were phosphodiesterase type 5 inhibitors (98 patients [62.4%]).

**Table 1:** Patient Characteristics and Examination Results at Baseline Right Heart Catheterization

Characteristic	Value
Age, y	53.1 ± 17.6
Female sex	111 (70.7)
BMI, kg/m^2^	21.8 ± 4.5
WHO FC III or IV	73 (46.5)
Smoking status, BI score	340 ± 548
Oxygen therapy	91 (58.0)
Lung parenchymal change	...
None to minimal	100 (63.7)
Intermediate	22 (14.0)
Extensive	35 (22.3)
PH subgroups	...
1	89 (56.7)
1 plus 3	27 (17.2)
3	41 (26.1)
No. of PAH therapies	...
0	27 (17.2)
1	53 (33.8)
2	31 (19.7)
3	46 (29.3)
Type of PAH therapy	...
Oral prostacyclin	52 (33.1)
Parenteral prostacyclin	16 (10.2)
ERA	78 (49.7)
PDE5-i	98 (62.4)
sGC-s	9 (5.7)
Blood test results	...
Hemoglobin, g/dL	13.7 ± 2.4
UA, mg/dL	5.7 ± 1.9
BNP, pg/mL	141 ± 178
PFT results	...
FVC, % predicted	78.7 ± 23.6
FEV_1_, % predicted	71.6 ± 22.6
Dlco, % predicted	54.2 ± 24.3
6MWT (n = 127)	...
Distance, m	365 ± 115
Modified Borg scale, median	4
Minimum Spo_2_, %	82 ± 10
UCG (n = 137)	...
TAPSE, mm)	18.6 ± 4.4
TAM-S′, cm/s	11.5 ± 3.3
Pericardial effusion	33 (24.1)
RHC	...
RAP, mm Hg	5.0 ± 4.7
mPAP, mm Hg	40.9 ± 12.2
PAWP, mm Hg	7.6 ± 3.2
Cardiac index, L/min/m^2^	3.01 ± 0.93
PVR, Wood unit	7.8 ± 4.0
Svo_2_, %	68.1 ± 7.6
Pao_2_, Torr	70.7 ± 26.1
Paco_2_, Torr	40.4 ± 8.3

Data are presented as No. (%) or mean ± SD unless otherwise indicated. 6MWT = 6-min walk test; BI = Brinkman index; BNP = brain natriuretic peptide; Dlco = diffusing capacity of the lungs for carbon monoxide; ERA = endothelin receptor antagonist; mPAP = mean pulmonary arterial pressure; PAH = pulmonary arterial hypertension; PAWP = pulmonary arterial wedge pressure; PDE5-i = phosphodiesterase 5 inhibitor; PFT = pulmonary function testing; PH = pulmonary hypertension; PVR = pulmonary vascular resistance; RAP = right arterial pressure; RHC = right heart catheterization; sGC-s = soluble guanylate cyclase stimulator; Spo_2_ = saturation of percutaneous oxygen; Svo_2_ = mixed venous oxygen saturation; TAM-S′ = tricuspid annular motion systolic velocity; TAPSE = tricuspid annular plane systolic excursion; UA = uric acid; UCG = ultrasound cardiography; WHO FC = World Health Organization functional class.

### Relationship Between %NLV and Clinical Findings

**Figure 3:**
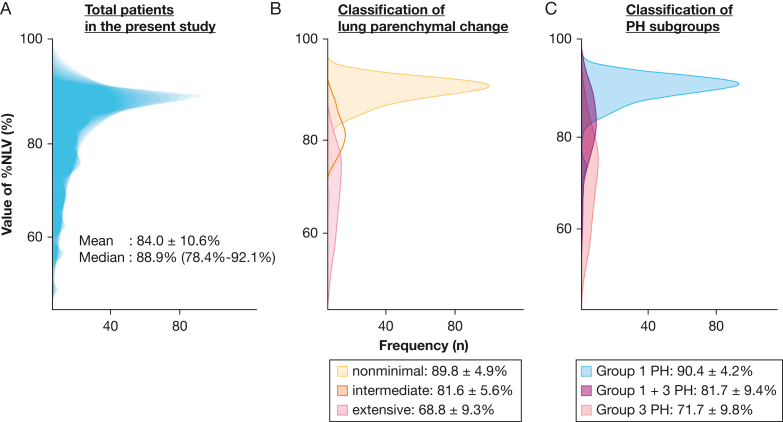
Histograms showing the distribution of %NLV. A, Histogram showing the distribution of %NLV in all patients. %NLV is presented using mean ± SD and median (interquartile range). B, Stratified histogram of %NLV according to the classification of lung parenchymal change on high-resolution CT imaging interpretation. %NLV in the three groups are presented as the mean ± SD. C, Stratified histogram of %NLV according to the classification of PH subgroups. %NLV in the three groups is presented as mean ± SD. %NLV = percentage of normal lung volume to the whole lungs; PH, pulmonary hypertension.

The histogram showing the distribution of %NLV measured using a 3-dimensional image analysis system based on the chest HRCT imaging of the patients is shown in Figure 3A. The mean and median %NLV were 84.0 ± 10.6% and 88.9% (interquartile range, 78.4%-92.1%). Figure 3B, 3C shows stratified histograms of %NLV, dividing patients into three groups based on the classification of lung parenchymal change on HRCT imaging and PH subgroups (for the relationship between lung parenchymal change and PH subgroups, see e-Table 1). As shown in Figure 3B, significant differences in %NLV were found among the three groups (nonminimal vs intermediate, *P < .*0001; nonminimal vs extensive, *P < .*0001; intermediate vs extensive, *P < .*0001). Similarly, significant differences were found in the %NLV of the three groups divided by PH subgroups, as shown in Figure 3C (group 1 vs group 1 plus group 3, *P < .*0001; group 1 vs group 3, *P < .*0001; group 1 plus group 3 vs group 3, *P < .*0001).

**Figure 4:**
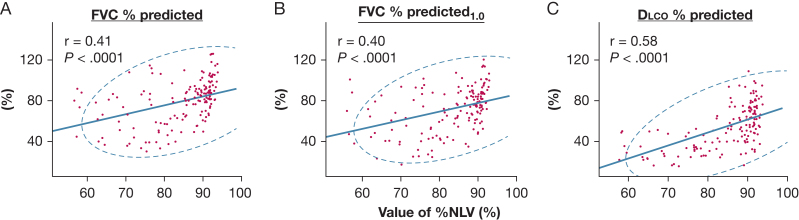
A-C, Scatterplots and regression lines showing the association percentage of NLV to the whole lungs (%NLV) with pulmonary function testing parameters: FVC % predicted (A), FEV_1_ % predicted (B), and Dlco % predicted (C). Correlation coefficients (r) and probability values (*P*) are presented in each figure. Dlco, diffusing capacity of the lungs for carbon monoxide.

The association between %NLV and PFT parameters is shown as a scatterplot in Figure 4. The correlation coefficients between %NLV and the PFT parameters such as FVC % predicted (Fig 3A), FEV_1_ % predicted (Fig 3B), and Dlco % predicted (Fig 3C) were 0.41 (*P < .*0001), 0.40 (*P < .*0001), and 0.58 (*P < .*0001), respectively.

### Survival Analysis With %NLV

**Figure 5:**
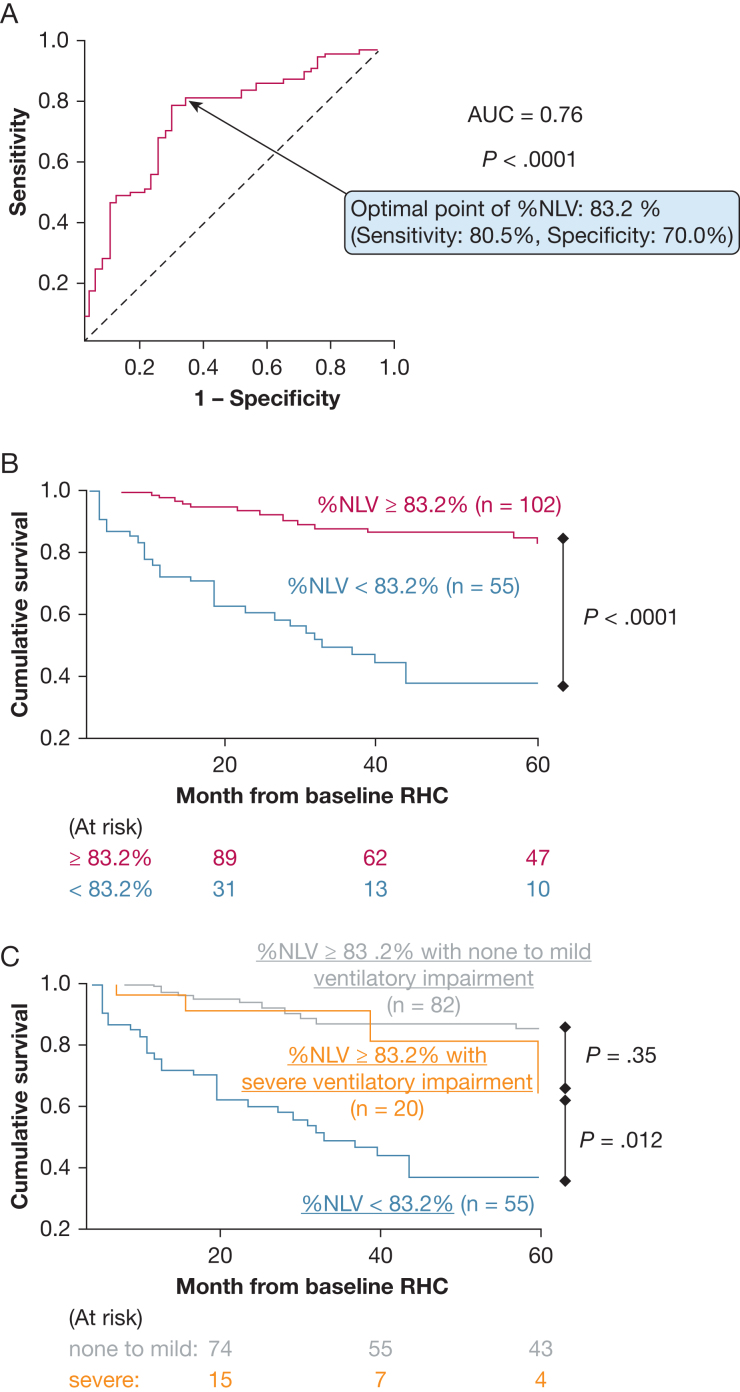
Survival analysis. A, Receiver operating characteristic curve obtained by logistic regression analysis to estimate the relationship between the %NLV and 3-year survival. The arrow point indicates the optimal cutoff of %NLV for 3-year survival. B, Comparison of Kaplan-Meier curves of 5-year survival between patients with %NLV of ≥ 83.2% and < 83.2%. C, Kaplan-Meier curves showing patients with %NLV of ≥ 83.2% further divided into two subgroups according to the severity of ventilatory impairment and the survival curves. AUC = area under the receiver operating characteristic curve; %NLV = percentage of normal lung volume to the whole lungs; RHC = right heart catheterization.

In the survival analysis, 58 patients (36.9%) died during the follow-up period. The 1-year, 3-year, 5-year, and 10-year survival rates were 86.6%, 72.4%, 66.4%, and 51.9%, respectively. The ROC curve for the effect of %NLV on 3-year survival is shown in Figure 5A. The area under the ROC curve was 0.76 (*P < .*0001), and the optimal cutoff point of %NLV for 3-year survival was 83.2% (sensitivity, 80.5%; specificity, 70.0%). The Kaplan-Meier curve for the 5-year survival rates of the patients divided into two groups based on the %NLV cutoff point is shown in Figure 5B. The patients with %NLV of ≥ 83.2% (n = 102) showed a significantly better 5-year survival rate than that of those with %NLV of < 83.2% (n = 55; 81.7% vs 36.6%; *P < .*0001). In addition, when the patients with %NLV of ≥ 83.2% were divided further into two subgroups based on severe ventilatory impairment (Fig 5C), no significant difference was found in 5-year survival regardless of the presence or absence of severe ventilatory impairment (84.0% vs 64.0%; *P = .*35). However, the group with %NLV of ≥ 83.2% combined with severe ventilatory impairment showed significantly better 5-year survival than the group with %NLV of < 83.2% (64.0% vs 36.6%; *P = .*012).

In the univariate analysis, %NLV of < 83.2% (HR, 5.15 [95% CI, 2.81-9.46]; *P < .*0001), severe ventilatory impairment (HR, 2.77 [95% CI, 1.55-4.95]; *P = .*0006), median age of ≥ 57 years (HR, 1.91 [95% CI, 1.06-3.45]; *P = .*031), male sex (HR, 3.12 [95% CI, 1.76-6.11]; *P = .*0001), World Health Organization functional class III or IV (HR, 3.27 [95% CI, 1.75-6.11]; *P = .*0002), 6MWT distance of < 440 m (HR, 6.67 [95% CI, 1.59-28.02]; *P = .*0095), and mixed venous oxygen saturation of ≤ 65% (HR: 2.12 [95% CI, 1.16-3.85]; *P = .*014) were identified as significant prognostic factors. In the multivariable analysis (goodness of fit, *P < .*0001), %NLV of < 83.2% was the most significant prognostic factor (HR, 2.49 [95% CI, 1.14-5.44]; *P = .*022) (Table 2).

**Table 2:** Univariable and Multivariable Cox Proportional Hazards Regression Analyses

Factor	Univariable		Multivariable	
	HR (95% CI)	*P* Value	HR (95% CI)	*P* Value
%NLV	...	< .0001	...	.022
≥ 83.2%	1.00	...	1.00	...
< 83.2%	5.15 (2.81-9.46)	...	2.49 (1.14-5.44)	...
Ventilatory impairment	...	.0006	...	.14
None to mild	1.00	...	1.00	...
Severe	2.77 (1.55-4.95)	...	1.72 (0.84-3.51)	...
Median age, y	...	.031	...	.59
< 57	1.00	...	1.00	...
≥ 57	1.91 (1.06-3.45)	...	1.19 (0.62-2.28)	...
Sex	...	.0001	...	.011
Female	1.00	...	1.00	...
Male	3.12 (1.76-5.54)	...	2.17 (1.19-3.96)	...
WHO FC	...	.0002	...	.055
I or II	1.00	...	1.00	...
III or IV	3.27 (1.75-6.11)	...	1.93 (0.99-3.76)	...
BNP, pg/mL	...	.74	...	...
< 50	1.00	...	...	...
≥ 50	1.10 (0.62-1.98)	...	...	...
6MWT, m	...	.0095	...	...
≥ 440	1.00	...	...	...
< 440	6.67 (1.59-28.02)	...	...	...
Pericardial effusion	...	.91	...	...
Without	1.00	...	...	...
With	1.04 (0.51-2.12)	...	...	...
Cardiac index, L/min/m^2^	...	.066	...	.34
≥ 2.5	1.00	...	1.00	...
< 2.5	1.73 (0.96-3.10)	...	1.37 (0.72-2.63)	...
PVR, WU	...	.14	...	...
≤ 5	1.00	...	...	...
> 5	1.68 (0.84-3.39)	...	...	...
Svo_2_, %	...	.014	...	...
> 65	1.00	...	...	...
≥ 65	2.12 (1.16-3.85)	...	...	...

This table shows the significant prognostic factors in the univariate and multivariable Cox proportional hazards regression analyses. 6MWT = 6-min walk test; BNP = brain natriuretic peptide; HR = hazard ratio; %NLV = percentage of normal lung volume to the whole lungs; PVR = pulmonary vascular resistance; Svo_2_ = mixed venous oxygen saturation; WHO FC = World Health Organization functional class; WU = Wood unit.

### Response to Pulmonary Vasodilators and %NLV

The response to pulmonary vasodilators associated with %NLV was examined in 81 patients (51.6%). Among them, 53 patients (65.4%) were categorized as responders and 28 patients (34.6%) were categorized as nonresponders. The comparison of patient characteristics between the responders and nonresponders is shown in Table 3. The responders showed significantly higher %NLV than the nonresponders (90.0 ± 5.1% vs 84.7 ± 9.2%; *P = .*0002). The responders also were significantly younger (46.2 ± 16.6 years vs 54.1 ± 17.3 years; *P = .*036) and had higher FVC % predicted (87.6 ± 18.5% predicted vs 77.0 ± 19.1% predicted; *P = .*019), longer 6MWT distance (418 ± 102 m vs 344 ± 120 m; *P = .*018), and higher pulmonary vascular resistance (9.6 ± 4.2 Wood units vs 7.0 ± 3.5 Wood units; *P = .*0049) than those of nonresponders. Multivariable logistic regression analysis (goodness of fit, *P = .*0054) revealed that high %NLV (OR, 1.12 [95% CI, 1.01-1.23]; *P = .*016) was the only significant contributor to treatment response (Table 4).

**Table 3:** Comparison of Patient Characteristics Between Responders and Nonresponders to Pulmonary Vasodilators

Characteristic	Responder (n = 53)	Nonresponder (n = 28)	*P* Value
%NLV, %	90.0 ± 5.1	84.7 ± 9.2	.0002
Age, y	46.2 ± 16.6	54.1 ± 17.3	.036
Female sex	41 (77.4)	24 (85.7)	.36
WHO FC III or IV	21 (39.6)	10 (35.7)	.73
BNP, pg/mL	139 ± 186	167 ± 174	.16
FVC, % predicted	87.6 ± 18.5	77.0 ± 19.1	.019
FEV_1_, % predicted	78.7 ± 17.4	72.2 ± 18.8	.17
6MWT distance, m	418 ± 102	344 ± 120	.018
Pericardial effusion	11 (24.4)	6 (26.1)	.88
Cardiac index, L/min/m^2^	2.96 ± 1.03	3.07 ± 0.90	.30
PVR, WU	9.6 ± 4.2	7.0 ± 3.5	.0049
Svo_2_, %	69.7 ± 7.9	68.5 ± 5.3	.51

The clinical differences between responders and non-responders are assessed using Mann–Whitney *U* test or chi-square test. 6MWT = 6-min walk test; BNP = brain natriuretic peptide; %NLV = percentage of normal lung volume to the whole lungs; PVR = pulmonary vascular resistance; Svo_2_ = mixed venous oxygen saturation; WHO FC = World Health Organization functional class; WU = Wood unit.

**Table 4:** Multivariable Logistic Regression Analysis for Factors Associated With Response to Pulmonary Vasodilators

Factor	OR (95% CI)	*P* Value
%NLV, %	1.12 (1.01-1.23)	.016
Age, y	0.98 (0.95-1.01)	.21
WHO FC III or IV	2.13 (0.66-6.83)	.19
FVC, % predicted	1.01 (0.98-1.04)	.42

%NLV = percentage of normal lung volume to the whole lungs; WHO FC = World Health Organization functional class.

## Discussion

To our knowledge, no evidence-based guidelines have been established to distinguish between group 1 and group 3 PH in patients with PH accompanied by parenchymal lung diseases. However, some researchers have reported that quantitative CT imaging could have potential clinical benefits for phenotyping.^19^ Although a quantitative CT imaging analysis in patients with PH has been performed in several studies,^20^, ^21^, ^22^ to our knowledge, this study is the first to evaluate quantitative NLV on chest CT imaging in patients with PH using a 3-dimensional image analysis system. We found that %NLV was a clinically important marker in patients with physician-diagnosed group 1 PH and group 3 PH. Therefore, evaluating %NLV via 3-dimensional image analysis system potentially could be useful to distinguish between groups 1 and 3 PH in patients also demonstrating parenchymal lung diseases.

A novel aspect of this study is that it focused on the NLV of patients with PH as an important clinical indicator. Ninagawa et al^23^ showed that the calculated abnormal lung volume measured using a 3-dimensional analysis system predicted the response to vasodilators in patients with systemic sclerosis combined with PH. However, in patients with PH with concurrent parenchymal lung diseases, the area that appears normal on HRCT imaging may have vascular remodeling expected to respond to pulmonary vasodilators, similar to PAH. In contrast, patients with an abnormal area on HRCT imaging would not respond to pulmonary vasodilators because of structural changes in the lungs, resulting in decreased vascular beds. Moreover, when emphysema and fibrosis coexist in abnormal areas, it is extremely difficult to evaluate lung damage related to PH comprehensively. Based on these findings, the degree of NLV is a more appropriate marker when treating patients with PH accompanied by parenchymal lung diseases.

In the present study, %NLV was identified as a significant independent prognostic factor in patients with PH, consistent with the results of the Assessing the Spectrum of Pulmonary hypertension Identified at a REferral centre (ASPIRE) registry.^12^ Although visual interpretation of chest CT scans has been used to determine the presence or absence of lung diseases in the ASPIRE registry, quantitative CT scan analysis was conducted to measure %NLV as an assessment of lung conditions in the present study. On objective evaluation of lung condition using quantitative CT imaging with a 3-dimensional image analysis system, patients with %NLV of ≥ 83.2% showed significantly better 5-year survival than that of those with %NLV of < 83.2% (81.7% vs 36.6%; *P < .*0001), suggesting that even a relatively small reduction in %NLV has a significant impact on the prognosis of patients with PH.

In a subgroup analysis of patients with PH and %NLV of ≥ 83.2%, prognosis was not associated with severe ventilatory impairment in PFT. This result is inconsistent with a report of a large Japanese registry that investigated patients with PH associated with lung diseases.^18^ This might be explained by the difference in study patients. The present study intentionally included patients with no or subtle lung parenchymal changes on chest CT imaging, whereas the Japanese registry involved only patients with evident lung diseases (group 3 PH). Moreover, severe ventilatory impairment is not always caused by parenchymal lung disease. A previous study reported that patients with group 1 PH showed lower FVC % predicted and FEV_1_ % predicted than healthy people because of peripheral airway obstruction involved in inflammation caused by the remodeling of the peripheral pulmonary artery.^24^^,^^25^ The present study showed that the %NLV in patients with PH might be a more objective prognostic marker than ventilatory impairment, especially in patients with subtle lung parenchymal disease.

This study showed that %NLV might be a useful predictor of response to pulmonary vasodilators in patients with PH. This could be explained by Dlco % predicted, an important marker for PFT. Dlco % predicted can assess the amount of vascular bed in patients with PAH^26^ and is considered a prognostic factor.^27^ Dlco % predicted also recently was reported to be correlated with the parameters of quantitative CT imaging in COPD.^28^ In the present study, Dlco % predicted was correlated significantly with %NLV, and the level of Dlco % predicted and %NLV showed similar numerical trends in group 1 PH, group 1 plus group 3 PH, and group 3 PH (e-Table 2). Considering the association between Dlco % predicted and the amount of vascular bed, our results suggest that %NLV might reflect the extent of vascular lesions and Dlco % predicted and may identify responders to pulmonary vasodilators.

This study has some limitations. First, this was a single-center, retrospective, observational study; however, 1,471 consecutive patients who underwent RHC were included to avoid selection bias as much as possible. Future prospective multicenter studies using quantitative CT imaging and involving patients of different races and nationalities are necessary to universalize and clarify the significance of %NLV in patients with PH. Second, the patients had physician-diagnosed group 1 PH and group 3 PH, and the optimal cutoff point of %NLV might differ according to the subgroup patient ratio. Similarly, %NLV might be affected by the distribution of patients according to the severity of parenchymal lung change. Therefore, appropriate patient proportions by subgroup and severity of parenchymal lung change would be preferred for the statistical analysis, and more universal significance of %NLV in patients with PH might exist. Third, the long selection period of 20 years affected the prognosis and response to pulmonary vasodilators. Because treatments with pulmonary vasodilators in PH have advanced markedly,^3^ the difference in available pulmonary vasodilators from 2000 to 2020 may influence patient prognoses. Fourth, all patients were selected according to the Sixth World Symposium on Pulmonary Hypertension criteria. Although this choice could avoid missing patients with mild PH, it may have led to overtriaging of them, especially patients who received a diagnosis from 2000 through 2018. In light of these points, a prospective study with matched diagnostic criteria and treatment options needs to be conducted.

## Interpretation

Quantitative CT imaging analysis might allow numerical quantification of the lung condition and vasodilator treatable area beyond subjective visual assessment in patients with PH. Therefore, %NLV could be a novel predictor of prognosis and treatment response. Further studies using artificial intelligence approaches have the potential to reveal the significance of the NLV and to provide novel insights into PH.

## Funding/Support

This study was supported in part by the Intractable Respiratory Diseases and Pulmonary Hypertension Research Group and the Ministry of Health, Labor and Welfare of Japan.

## Financial/Nonfinancial Disclosures

None declared.

## Supplementary Data

e-Online Data
