# Supplementary material for: Significance of Normal Lung Volume on Quantitative CT Imaging Analysis in Group 1 and Group 3 Pulmonary Hypertension
Source: CHEST Pulm. 2024 May 11;2(4):100062. doi: 10.1016/j.chpulm.2024.100062 (PMC13418607; doi:10.1016/j.chpulm.2024.100062)
Supplement: e-Online Data [file mmc1.docx]

**SUPPLEMENTAL MATERIALS**

e-Table 1: Relationship between lung parenchymal change and PH subgroups

|  | Lung parenchymal change | | |  |
| --- | --- | --- | --- | --- |
|  | None-minimal  (n=100) | Intermediate  (n=22) | Extensive  (n=35) | |
| PH subgroups |  |  |  | |
| Group 1 PH (n=89) | 89 | 0 | 0 | |
| Group 1+3 PH (n=27) | 8 | 12 | 7 | |
| Group 3 PH (n=41) | 3 | 10 | 28 | |
|  |  |  |  | |

This table shows the relationship between the classification of lung parenchymal change on high-resolution computed cosmography (HRCT) and pulmonary hypertension (PH) subgroups with clinical characteristics. The none-minimal group was mainly categorized as Group 1 PH, while the extensive group as Group 3 PH. Patients with Group 3 PH who had none to minimal change (n=3) had non-emphysematous chronic obstructive pulmonary disease. The intermediate group involved half of Group 1+3 PH patients and half of Group 3 PH patients.

HRCT, high-resolution computed tomography; n, number; PH, pulmonary hypertension

e-Table 2: Patient characteristics by PH subgroup

| Characteristic | Group 1 PH  (n=89) | Group 1+3 PH  (n=27) | Group 3 PH  (n=41) |
| --- | --- | --- | --- |
| Age (years) | 46.2±17.3 | 59.6±15.2 | 63.8±12.3 |
| Female sex, n (%) | 76 (85.4) | 19 (70.4) | 16 (39.0) |
| BMI (kg/m^2^) | 22.2±4.0 | 21.8±5.2 | 21.1±5.1 |
| WHO-FC Ⅲ/Ⅳ, n (%) | 31 (34.8) | 11 (40.7) | 31 (75.6) |
| Smoking status (BI score) | 133±256 | 346±664 | 813±657 |
| Oxygen therapy, n (%) | 38 (42.7) | 16 (59.3) | 36 (87.8) |
| %NLV |  |  |  |
|  | 90.4±4.2 | 81.7±9.4 | 71.7±9.8 |
| PFT |  |  |  |
| %FVC (%) | 89.0±16.3 | 66.5±24.2 | 64.3±25.9 |
| %FEV_1.0_ (%) | 81.8±15.7 | 61.9±19.9 | 55.9±25.4 |
| %DL_CO_ (%) | 65.9±22.0 | 44.2±17.9 | 33.3±14.1 |
| RHC |  |  |  |
| RAP (mmHg) | 6.1±5.5 | 4.3±2.4 | 3.1±3.0 |
| mPAP (mmHg) | 45.1±12.2 | 37.0±10.7 | 34.4±9.2 |
| PAWP (mmHg) | 7.9±3.2 | 7.8±2.8 | 6.6±3.3 |
| CI (L/min/m^2^) | 3.08±0.93 | 3.07±0.92 | 2.83±0.92 |
| PVR (wood units) | 8.6±4.3 | 6.7±3.6 | 6.7±3.3 |
| Numbers of PAH therapy |  |  |  |
| None, n (%) | 8 (9.0) | 2 (7.4) | 17 (41.5) |
| Monotherapy, n (%) | 23 (25.8) | 11 (40.8) | 19 (46.3) |
| Double therapy, n (%) | 19 (21.4) | 9 (33.3) | 3 (7.3) |
| Triple therapy, n (%) | 39 (43.8) | 5 (18.5) | 2 (4.9) |
|  |  |  |  |

This table shows patients characteristics classified by PH subgroups: Group 1 PH, Group 1+3 PH, and Group 3 PH. Patients with Group 1+3 PH exhibit intermediate characteristics between Group 1 PH and Group 3 PH in each category.

%DL_CO_, percent predicted diffusing capacity of the lung for carbon monoxide; %FEV_1.0_, percent predicted forced expiratory volume in 1 second; %FVC, percent predicted forced vital capacity; BI score, Brinkman index score; BMI, body mass index; CI, cardiac index; mPAP, mean pulmonary arterial pressure; n, number; NLV, normal lung volume; PAH, pulmonary arterial hypertension; PAWP, pulmonary arterial wedge pressure; PFT, pulmonary function test; PH, pulmonary hypertension; PVR, pulmonary vascular resistance; RAP, right arterial pressure; RHC, right heart catheterization; WHO-FC, The World Health Organization functional classification
